# Retrospective Study of Oral Lichen Planus and Oral Lichenoid Lesions: Clinical Profile and Malignant Transformation 

**DOI:** 10.30476/DENTJODS.2021.91356.1572

**Published:** 2022-12

**Authors:** Shahla Kakoei, Molook Torabi, Maryam Rad, Nahid Karbasi, Sahar Mafi

**Affiliations:** 1 Dept. of Oral Medicine, Oral and Dental Diseases Research Center, Kerman University of Medical Sciences, Kerman, Iran; 2 Dept. Oral Pathology, Social Determinants on Oral Health Research Center, Kerman University of Medical Sciences, Kerman, Iran; 3 Social Determinant on Oral Health Research Center, Kerman University of Medical Sciences, Kerman, Iran; 4 Dept. of Oral Medicine, School of Dentistry, Semnan University of Medical Sciences, Semnan, Iran; 5 Dept. Oral Medicine, School of Dentistry, Kermanshah University of Medical Sciences, Kermanshah, Iran

**Keywords:** Oral Lichen Planus, Oral Lichenoid Lesion, Dysplasia, Squamous Cell Carcinoma, Malignant Transformation

## Abstract

**Statement of the Problem:**

Oral lichen planus (OLP) and other oral lichenoid lesions (OLL) are reported to have the potential of malignant transformation and dysplastic changes, turning into oral squamous cell carcinoma (SCC). While the world health organization (WHO) has classified OLP as a precancerous lesion of the oral cavity, there is still much debate among researchers about its risks and malignancy potential.

**Purpose:**

The present study aimed to determine malignant transformation in OLP and OLL and understand related risk factors.

**Materials and Method:**

This retrospective study was performed on 356 patients of the Oral Medicine Department of Dental School of Kerman Medical University from 1998 to 2020. All patients’ records were gathered. In addition, patients were followed up routinely. Second biopsy was taken as needed. The samples, previously taken from the patients, were re-evaluated according to WHO histopathologic criteria for diagnosing OLP, OLL, dysplasia, and SCC by an experienced pathologist and compared with first reports.

**Results:**

Dysplastic changes were observed in 6.2% of the patients. In more than half of the patients, dysplastic changes were present right from the start and 2.20% of the patients had experienced dysplastic changes averagely within 2.05 years of the onset of lesions. Multiple logistic regression showed that the risk of dysplasia increases with aging (p= 0.013), smoking (p= 0.0001), and thyroid disorders (p= 0.008).

**Conclusion:**

Given the rather high prevalence of oral lichen planus and lichenoid lesions, further research appears to be needed to determine the etiology of these lesions, malignant transformations, and the factors affecting this probability. Considering the findings, it is imperative to meticulously record the information of all patients with oral lichen planus and lichenoid lesions in the initial examinations as well as close follow-ups and employ diagnostic tools such as toluidine blue staining or even repeat biopsy when necessary.

## Introduction

Oral lichen planus (OLP) is a common chronic inflammatory mucocutaneous disorder. This disorder usually affects the oral mucosa, but can also occur on the skin, genital mucosa, scalp, and nails [ 1 ]. The global prevalence of OLP has been estimated at 0.5-2.6%. Research has shown that the mean age of onset of OLP is 50-60 years and it is more common in women than in men by a ratio of 3 to 2 [ 1 ]. OLP lesions manifest as bilateral white keratotic lines with reticular and radial patterns (Wickham striae) in an atrophic or erosive bed [ 1 ].

There is another group of oral lesions with almost the same clinical and histopathological characteristics as OLP, which are called oral lichenoid lesions (OLL) or oral lichenoid reactions (OLRs). These include contact hypersensitivity to dental materials, drug-induced lichenoid lesions, lichenoid reactions in chronic graft-versus-host disease, and lichenoid dysplasia [ 1 ]. Histopathological examinations alone are not enough to differentiate between OLLs and OLP reliably. For this differentiation, one has to also examine the clinical characteristics of the lesions and make a diagnosis based on the diagnostic criteria provided by the world health organization (WHO) in 2003. However, even with these criteria, it is sometimes impossible to differentiate these lesions [ 2 ].

One of the most important issues regarding OLP is the potential for malignancy and the possibility of dysplastic changes and conversion to oral squamous cell carcinoma (SCC). Although the WHO has classified OLP as a potentially malignant condition, there is still much debate among researchers about the malignancy potential and risk of OLP [ 3 ]. The mechanism that causes OLP to become malignant is not exactly known. It has been suggested that chronic inflammation in these lesions and inflammatory mediators can lead to genetic changes in the epithelium that accelerate malignant transformation [ 4 ].

Some researchers have suggested that only OLLs, and not OLP, are malignant and should be classified as lichenoid dysplasia [ 5 ]. However, there have been numerous reports of SCC in patients who have previously been diagnosed with OLP without malignant transformation. In addition, there have been numerous reports of multifocal dysplasia and multifocal SCC in patients with OLP, which indicate the possibility of field cancerization in OLP [ 1 , 3 , 6 ].

It is reported that some OLP lesions that turned into SCC may have been reported as OLP mistakenly, and dysplasia with lichenoid patterns should have been excluded from the study [ 2 , 7 ].

To address the problem of insufficient information for classifying OLP lesions that showed malignant transformation, in 2003, WHO introduced a new classification for the differentiation of OLP and OLLs, including lichenoid dysplasia lesions [ 5 ]. 

In recent years, most follow-up studies have shown the potential of OLP lesions to turn into malignancy [ 1 , 5 - 6 , 8 - 11 ]. According to recent studies, the risk of malignant transformation in OLP is 1 to 2% [ 1 , 10 , 12 - 14 ]. Oral cancers are the eighth leading cause of death from cancer, but many patients are unaware of malignant changes in their oral cavities. Early detection of oral epithelial dysplasia and malignant changes can prevent many deaths from oral cancers and provide an opportunity to limit the progression of lesions and their subsequent complications through non-invasive treatments [ 15 ].

To date, many detailed epidemiological and clinical studies of OLP have been undertaken [ 8 - 9 , 16 - 17 ]. The results of these studies help clinicians gain a better perception of OLLs and related factors and open up new horizons for future studies. The present retrospective study aimed to determine the age and sex, clinical presentation, symptoms, systemic factors, the predisposing and aggravating factors, complete blood count (CBC) findings and malignant transformation in OLP and OLL patients of Oral Medicine Department of Dental School of Kerman Medical University in the past 10 years (1998-2020).

## Materials and Method

This cross-sectional retrospective study was performed on OLP and OLL patients of the Oral Medicine Department of Dental School of Kerman Medical University. Following the approval of the University’s Ethics Committee (IR.KMU.REC.1396.2160), the records of all patients of the Oral Medicine Department from April 1997 to December 2019 were searched for diagnoses of OLP or OLLs. After finding the records of patients diagnosed with OLP or OLLs, the demographic data, the history of systemic diseases and medication, the test results at the time of diagnosis, the patient’s habits such as smoking, drug use, opium use, and alcohol use, clinical findings such as lesion site, presence of erythema and ulcers, and so on were collected.

The samples which were previously taken from the patients re-evaluated according to WHO histopathologic criteria for diagnosing OLP, OLL, dysplasia and SCC by an experienced pathologist; each histopathologic finding were recorded in a checklist based on WHO criteria and diagnostic decision was made then the result was compared with first report. The pathologist was unaware of the previous diagnosis [ 9 ]. Patients were called for follow-up. For cases with suspicious oral lesions such as white plaques or white and red lesions with a lichenoid pattern along with ulcers and erythema, the toluidine blue staining test was performed, and if positive, samples were taken and histopathologic evaluation based on WHO criteria were done while appropriate treatments were carried out. The results of histopathology, the clinical characteristics observed in the follow-up session, and changes in demographic information, history, and habits were recorded in the checklist.

The patients whose first clinical diagnosis had not been confirmed by biopsy and histological examination, the patients who did not attend the follow-up appointment, and the patients who did not agree to repeat the biopsy (if necessary) were excluded from the study. The patients who had received laser therapy, cryotherapy, or vitamin Aderived medications before the follow-up in a way that had altered the appearance of the lesions were also excluded from the study. The collected data were analyzed by the T-test, chi-square test, and logistic regression in SPSS v.21. In these analyses, *p* Value < 0.05 was considered statistically significant.

## Results

The study identified 356 patients with a final diagnosis of OLP or OLL from April 1997 to December 2020. According to WHO criteria for diagnosing OLP and OLL, 82.6% had OLP and 17.4% had OLL. The mean age of the patients at the time of initial diagnosis was 47±14.2. Of the 354 patients (2 patients were missing), 97 (27.4%) were male and 257 (72.6%) were female. Of these 354 patients, 196 patients (55.1%) had no systemic disease. Among those who had systemic diseases, thyroid diseases (9.6%) , hypertension (8.1%), and diabetes (4.8%) were the most common. Of the total population of patients, 34% were taking medication. The most common medications were heart and blood pressure medications at 63.5% (80 out of 126), diabetes medications at 21.4% (27 out of 126), and thyroid medications at 19.8% (25 out of 126) (Table 1). In addition, 12.1% of the patients reported being smokers or users of alcohol or drugs. Among this group, smoking and opium use were the most common habits). Clinically, more than half of the lesions were multifocal and the most common lesion site was the buccal mucosa (Table 2). Dysplastic changes were observed in 6.2% of the patients (22 patients: 14 females (63.6%) and 8 males (36.4%)). All the malignant transformations found in the same place as previously were affected by lichen planus lesions. In patients with multiple sites affected by lesions at least one site showed malignant transformation. Re-examination of the archived samples based on WHO criteria showed that for more than half of the patients, dysplastic changes were present right from the start. In 2.2% of cases, a malignant transformation had occurred over time. The mean time before the occurrence of dysplastic changes was 2.05 years and the mean age of patients at the onset of dysplastic changes was 53 years. All malignancies were related to OLP lesions and there was no dysplasia in OLL patients. In 9 of these patients (40.9%), the malignant transformation was in the form of well-defined squamous cell carcinoma, and in 13 of them (59.1%) it was in the form of mild to severe dysplasia. The most common sites of malignant transformation were the lateral border of the tongue, the buccal mucosa, and the ventral surface of the tongue (Table 3). Multiple logistic regression showed that aging (p= 0.013), smoking (p= 0.0001), and abnormal thyroid (p= 0.008) increase the risk of dysplasia.

**Table 1:** Frequency distribution of patients by sex, history of systemic diseases, drug use, blood test results and type of lesions

	N.	%
Sex		
Female	257	72.60
Male	97	27.40
Systemic illness		
ttttDiabetes	17	4.80
Hypertension	29	8.10
Thyroid anomalies	34	9.60
Hypothyroidism	31	91.18
Hyperthyroidism	3	8.82
No systemic diseases	196	55.10
Blood test		
ttttAnemia	46	12.90
High FBS*	19	5.30
TSH** anomalies	8	2.30
LFT*** anomalies	19	5.30
High blood lipids	23	6.50
Normal	241	67.70
Type of lesion		
ttOLP	294	82.60
OLL	62	17.40

*FBS: Fasting Blood Suger

** TSH: Thyroied stimulating hormone

*** LFT: Liver Function Test

**Table 2:** Frequency distribution of lesions by site

Clinical type	N.	%	Most common site
Unifocal58	58	16.3	Buccal
Bilateral	110	30.9	Buccal
Multifocal	188	52.8	Buccal, Tongue, Labial
Total	356	100	-

**Table 3:** Distribution of patients with malignant transformation by sex, type of lesion, and type of malignant changes over time

Malignant transformation	N.	%
Female	14	72.6
Male	8	27.40
In OLP*	22	100
In OLL**	0	0
Histologic degree of dysplasia		
ttSCC***	9	40.90
Mild to severe dysplasia	13	59.09

*Oral lichen planus

** Oral lichenoid lesion

*** Squamous cell carcinoma

## Discussion

The study investigated the malignant transformation of OLP lesions and its related factors in 365 patients of the Oral Medicine Department of Dental School of Kerman Medical University between 1998 and 2020.

The mean age of patients in this study (47±14.2) was similar to that reported by Li *et al.*[ 18 ]. In our study, 72.6% of the patients were women. The higher prevalence of OLP among women has also been reported in several other studies including Ingafou *et al.* (63.6%) [ 19 ], Ritcher *et al.* (72.5%) [ 20 ], and Pakfetrat *et al.* [ 9 ] (64.9%).

Among the population of this study, the most common systemic diseases were thyroid (9.6%), hypertension (8.1%), and diabetes (4.8%), respectively. In a cross-sectional study by Tang *et al.* [ 21 ] on the relationship between OLP and thyroid disorders, the prevalence of thyroid disease in OLP patients was reported to be 32.48%. They also recommended thyroid-screening tests for women with OLP. Pavan Kumar *et al.* [ 22 ] attributed the high prevalence of thyroid in OLP patients to autoimmunity problems. In the present study, thyroid disorder was the most common systemic disease among patients with OLP. It is not clear whether this relationship is due to a direct link between thyroid disorders and lichen planus lesions or is related to patients’ medications, an issue that should be further explored in future studies. If proven, this relationship can be used to identify patients with undetected thyroid problems.

At present, there is still no proof of an association between systemic diseases and OLP, as the findings of different studies in this area need to be further scrutinized to establish consistency. So far, OLP has been most commonly linked to diabetes mellitus and hepatitis C, but there is not enough evidence to prove such association, and no such association was observed in this study [ 23 - 24 ]. In the present study, laboratory tests showed that 12.9% of the patients had iron deficiency anemia. Considering that iron deficiency anemia is prevalent in women of reproductive age, this finding is not unexpected [ 25 ].

In a study carried out by Chen *et al.* [ 26 ] on the relationship between iron deficiency anemia, folic acid, B12 and OLP, 10.2% of OLP patients had iron deficiency anemia, which is close to the rate observed in this study. In another study, Sun *et al.* [ 27 ] reported that 13.6% of their OLP patients had iron deficiency anemia. They attributed this finding to the painful nature of oral lesions, which reduces food intake, and the prevalence of malnutrition and undernourishment in the age group of 53-57 and older. In a study by Alsheikh *et al.* [ 28 ] on the prevalence of oral lesions in anemic patients, 0.6% of these patients had OLP. In another study, Falsafi *et al.* [ 29 ] reported low levels of salivary transferrin in people with OLP and suggested the possibility of disposition to anemia in these individuals. Overall, further research is needed to prove a link between different types of anemia and OLP lesions. In this regard, the results of this study can be helpful in determining the etiology of the lesions and diagnosing undetected anemia.

In our study, 52.8% of the patients (188 individuals) had multifocal mucosal involvement, and the most common sites of lesions were buccal mucosa, tongue (ventral, and lateral border), and labial mucosa. In a study conducted by Gümrü *et al.* [ 11 ], lesions of about half of the subjects (47.6%) were multifocal, and similar to the present study, the most common site of lesions was buccal mucosa.

The preliminary results of this study showed dysplastic and carcinomatous changes in 6.2% of the patients. While dysplastic changes in OLLs and OLP lesions have been the subject of many studies, many researchers still do not agree on the extent of these changes over time. This in itself highlights the importance and necessity of further research in this area.

In a study by Gopalakrishnan *et al.* [ 30 ] on malignant transformations in patients with OLP over a 20-year period, the overall rate of malignancy was reported to be 1.25%. In a review and meta-analysis study by Idrees *et al.* [ 31 ] on the malignant transformations of OLP, they reported a malignancy rate of 1.1-1.4% for these lesions. Idrees *et al.* [ 31 ] stated that the reported malignancy rates for OLP are exaggerated and attributed it to how patients are chosen and the conditions of the studies. In addition, Iocca O *et al.* [ 32 ] and Giuliani *et al.* [ 33 ] reported same results as Idrees *et al.*’s study [ 31 ]. Some researchers believe that many of the lesions that suddenly show dysplastic or malignant changes in follow-ups have in fact been misdiagnosed as OLP or OLL because the original examination has missed the evidence of dysplasia [ 3 , 5 - 6 ]. This is why WHO has published its own diagnostic criteria for differentiating OLP and OLLs from lichenoid dysplasia [ 2 ]. In the present study, dysplastic changes were present or had developed over time in the original biopsy of 6.2% of patients. Once the archived samples used in original biopsies were re-examined based on the WHO criteria by an experienced pathologist, it was found that 50% of the cases that had been diagnosed as lichen planus had presented dysplastic changes right from the start. With this taken into account, the actual rate of malignant transformation in OLP lesions was 2.2%, which is still significantly higher than the figures reported in similar studies. The changes were in the form of well-defined SCC (40.9%) and mild to severe dysplasia (59.1%). The mean duration of these changes was 2.05±3.40 years. In a study by Shearston *et al.* [ 34 ], the mean time of occurrence of malignant transformation in a group of OLP patients in Italy was 4.8 years, which is longer than the corresponding time in the present study.

In this study, the most common sites of malignant transformation were the lateral border of the tongue, the buccal mucosa, and the ventral surface of the tongue. A study by Lanfranchi *et al.* [ 35 ] also identified the tongue as the most common site of malignancy in OLP patients. The lateral border of the tongue and the buccal mucosa are the most commonly reported sites of the transformation of OLP to squamous cell carcinoma. However, epithelial dysplasia in OLP lesions has been reported more frequently in the buccal mucosa [ 6 , 10 , 12 - 14 ].

In our study, dysplastic changes were observed in 22 patients (6.2%), of whom 14 were women (63.6%) and 8 were men (36.4%). The mean age of these patients at the time of dysplastic changes was 53 years. The findings of this study showed that dysplastic changes were significantly associated with aging (p= 0.013). However, in a meta-analysis conducted by Gonzalez *et al.* [ 36 ], they reported the same frequency for malignant transformations occurring before and after age 40. In contrast, Aghahoseyni *et al.* [ 37 ] reported that typically, oral cancers from OLP emerge when people are in their 60s and 70s. As in the present study, the meta-analysis of Gonzalez *et al.* [ 36 ] reported a higher prevalence of dysplastic changes in women, but this could be due to the higher overall prevalence of OLP in women, as the difference between these prevalence rates were not significant.

The data collected from the patients showed that the risk of dysplasia in OLP patients was significantly associated with smoking. However, in a study by Fang *et al.* [ 38 ], only 50% of OLP patients with malignant transformations had a history of drug and alcohol use, which was not statistically significant. In addition, in a review study by Giuliani *et al.* [ 33 ], the comparisons made between the populations of smokers and non-smokers did not show a significant positive relationship between smoking and the prevalence of malignant transformation in lichen planus.

In our study, patients with thyroid disorders had significantly higher malignancy rates (p=0.008). As mentioned earlier, none of the previous studies that have examined the potential relationship between thyroid disorders and OLP has reported such association. Since our findings contradict previous findings in this respect, further studies are needed to determine the reasons for this discrepancy. 

Many previous studies have reported the increased risk of dysplastic and malignant transformations in OLLs [ 31 - 33 ]. However, given the clinically and pathologically similar nature of OLP and OLL in many cases, they cannot be differentiated with certainty. In our study, the pathological examination conducted based on the WHO criteria showed that 100% of the observed dysplastic changes were related to OLP lesions; this finding contradicts the reports of several previous studies [ 5 - 6 ]. This finding is significant because some clinicians do not consider biopsies and close follow-ups to be necessary for lichen planus lesions, whereas the risk of dysplastic changes in these lesions was significant in this study.

In this study, we found no difference between different types of OLP in potential malignant transformations. In a study by Kaplan *et al.* [ 39 ], where more attention was paid to the dynamic and variable nature of OLP, it was stated that all types of OLP could undergo malignant transformations. Peng *et al.* [ 40 ], who studied the tumor-like microenvironments of OLP lesions, stated that the oxygen deficiency, inflammatory and immune characteristics, and acidity of these lesions could be the reasons for the higher prevalence of the development of OSCC.

## Conclusion 

Given the somewhat high prevalence of OLP and OLLs in the general population, further research is required to determine the etiology of these lesions, the chance of malignant transformations, and the factors that affect this probability. By creating a database of statistical information, these studies can contribute to the development of more efficient diagnostic criteria for OLP and OLLs, and better treatment protocols for patients with these conditions with attention to the risk of malignant transformation and the possibility of association with SCC and field cancerization. It is imperative to meticulously record the information of all OLP and OLL patients in the initial examinations and close follow-ups and use diagnostic tools such as toluidine blue staining or re-biopsy when necessary.

## Acknowledgement

This study was supported by Oral and Dental Diseases Research Center, Kerman University of Medical Sciences, Kerman, Iran. We would like to appreciate our colleagues in Oral Medicine Department and Oral Pathology Department of Dental School of Kerman University of Medical Sciences who provided expertise that greatly assisted the study.

## Conflict of Interest

The authors declare that they have no conflict of interest.

## References

[1] Alrashdan MS, Cirillo N, McCullough M ( 2016). Oral lichen planus: a literature review and update. Arch Dermatol Res.

[2] Van der Meij E, Van der Waal I ( 2003). Lack of clinicopathologic correlation in the diagnosis of oral lichen planus based on the presently available diagnostic criteria and suggestions for modifications. JOPM.

[3] Van der Meij EH, Schepman KP, van der Waal I ( 2003). The possible premalignant character of oral lichen planus and oral lichenoid lesions: a prospective study. Oral Surg Oral Med Oral Pathol Oral Radiol Endod.

[4] Peng Q, Zhang J, Ye X, Zhou G (2017). Tumor-like microenvironment in oral lichen planus: evidence of malignant transformation?. Expert Rev Clin Immunol.

[5] Van der Meij EH, Mast H, van der Waal I ( 2007). The possible premalignant character of oral lichen planus and oral lichenoid lesions: a prospective five-year follow-up study of 192 patients. Oral Oncol.

[6] Shirasuna K ( 2014). Oral lichen planus: Malignant potential and diagnosis. Oral Sci Int.

[7] Krutchkoff DJ, Cutler L, Laskowski S ( 1978). Oral lichen planus: the evidence regarding potential malignant transformation. JOP.

[8] Carbone M, Arduino PG, Carrozzo M, Gandolfo S, Argiolas MR, Bertolusso G, et al ( 2009). Course of oral lichen planus: a retrospective study of 808 northern Italian patients. Oral Dis.

[9] Pakfetrat A, Javadzadeh Bolouri A, Basir Shabestari S, Falaki F ( 2009). Oral Lichen Planus: a retrospective study of 420 Iranian patients. Med Oral Patol Oral Cir Bucal.

[10] Bombeccari GP, Guzzi G, Tettamanti M, Giannì AB, Baj A, Pallotti F, Spadari F ( 2011). Oral lichen planus and malignant transformation: a longitudinal cohort study. Oral Surg Oral Med Oral Pathol Oral Radiol Endod.

[11] Gümrü B ( 2013). A retrospective study of 370 patients with oral lichen planus in Turkey. Med Oral Patol Cir B.

[12] Lodi G, Scully C, Carrozzo M, Griffiths M, Sugerman PB, Thongprasom K ( 2005). Current controversies in oral lichen planus: report of an international consensus meeting Part 2 Clinical management and malignant transformation. Oral Surg Oral Med Oral Pathol Oral Radiol Endod.

[13] Lodi G, Scully C, Carrozzo M, Griffiths M, Sugerman P B, Thongprasom K ( 2005). Current controversies in oral lichen planus: report of an international consensus meeting Part 1 Viral infections and etiopathogenesis. Oral Surg Oral Med Oral Pathol Oral Radiol Endod.

[14] Yardimci G, Kutlubay Z, Engin B, Tuzun Y ( 2014). Precancerous lesions of oral mucosa. WJCC.

[15] Garg A, Chaturvedi P (2017). Prevention of oral cancer. Contemporary oral oncology.

[16] Rivera C, Jones Herrera C, Vargas P, Venegas B, Droguett D ( 2017). Oral diseases: a 14-year experience of a Chilean institution with a systematic review from eight countries. Med Oral Patol Cir B.

[17] Barbosa NG, Silveira EJ, Lima EN, Oliveira PT, Soares MS, de Medeiros AM ( 2015). Factors associated with clinical characteristics and symptoms in a case series of oral lichen planus. IJD.

[18] Li C, Tang X, Zheng X, Ge S, Wen H, Lin X, et al ( 2020). Global prevalence and incidence estimates of oral lichen planus: a systematic review and meta-analysis. JAMA Dermatology.

[19] Ingafou M, Leao J, Porter S, Scully C ( 2006). Oral lichen planus: a retrospective study of 690 British patients. Oral Dis.

[20] Richter I, Andabak Rogulj A, Vučićević Boras V, Brailo V ( 2014). Oral lichen planus–Retrospective study of 563 Croatian patients. Med Oral Patol Cir B.

[21] Tang Y, Shi L, Jiang B, Zhou Z, Shen X ( 2020). A cross-sectional study of oral lichen planus associated with thyroid diseases in east China. Front Endocrinol (Lausanne).

[22] Pavan Kumar T, Priyadharshini R, Sujatha S, Rakesh N, Shwetha V ( 2019). Association of OLP and thyroid disorder: Case report and review of literature. J Stomatol Oral Maxillofac Surg.

[23] Carrozzo M, Gandolfo S ( 2003). Oral diseases possibly associated with hepatitis C virus. Crit Rev Oral Biol Med.

[24] Otero Rey EM, Yáñez‐Busto A, Rosa Henriques IF, López‐López J, Blanco‐Carrión A ( 2019). Lichen planus and diabetes mellitus: Systematic review and meta‐analysis. Oral Dis.

[25] Sandvik J, Hole T, Klöckner CA, Kulseng BE, Wibe A ( 2020). intravenous iron treatment in the prevention of iron deficiency and anaemia after Rouxen-Y Gastric Bypass. Obes Surg.

[26] Chen HM, Wang YP, Chang JYF, Wu YC, Cheng SJ, Sun A ( 2015). Significant association of deficiencies of hemoglobin, iron, folic acid and vitamin B12 and high homocysteine level with oral lichen planus. JFMA.

[27] Sun A, Chang J, Chiang CP ( 2017). Examination of circulating serum autoantibodies and hematinics is important for treatment of oral lichen planus. JFMA.

[28] Alsheikh E, Amr E, Zahran F ( 2019). Prevalence of oral manifestations of iron deficiency anemia in a sample of egyptian population, hospital-based cross-sectional study. ADJC.

[29] Falsafi P, Khorshidi-Khiavi R, Ghanizadeh M, Rezaei F, Dolatkhah H, Bahramian A, et al ( 2018). Salivary transferrin levels in patients with oral lichen planus. PBOCI.

[30] Gopalakrishnan A, Balan A, Kumar NR, Haris P, Bindu P ( 2016). Malignant potential of oral lichen planus an analysis of literature over the past 20 years. Int J Appl Dent Sci.

[31] Idrees M, Kujan O, Shearston K, Farah CS ( 2021). Oral lichen planus has a very low malignant transformation rate: a systematic review and meta-analysis using strict diagnostic and inclusion criteria. J Oral Pathol Med.

[32] Iocca O, Sollecito TP, Alawi F, Weinstein GS, Newman JG, De Virgilio A, et al ( 2020). Potentially malignant disorders of the oral cavity and oral dysplasia: a systematic review and meta‐analysis of malignant transformation rate by subtype. Head & Neck.

[33] Giuliani M, Troiano G, Cordaro M, Corsalini M, Gioco G, Lo Muzio L, et al ( 2019). Rate of malignant transformation of oral lichen planus: A systematic review. Oral Dis.

[34] Shearston K, Fateh B, Tai S, Hove D, Farah CS ( 2019). Oral lich-enoid dysplasia and not oral lichen planus undergoes malignant transformation at high rates. JOPM.

[35] Lanfranchi Tizeira HE, Aguas SC, Sano SM ( 2003). Malignant transformation of atypical oral lichen planus: a review of 32 cases. Med Oral.

[36] González Moles MÁ, Ruiz Avila I, Gonzalez-Ruiz L, Ayen A, Gil-Montoya JA, Ramos-Garcia P ( 2019). Malignant transformation risk of oral lichen planus: a systematic review and comprehensive meta-analysis. Oral Oncol.

[37] Agha-Hosseini F, Sheykhbahaei N, SadrZadeh-Afshar M ( 2016). Evaluation of potential risk factors that contribute to malignant transformation of oral lichen planus: a literature review. J Contemp Dent Pract.

[38] Fang M, Zhang W, Chen Y, He Z ( 2009). Malignant transformation of oral lichen planus: a retrospective study of 23 cases. Quin Int.

[39] Kaplan I, Ventura Sharabi Y, Gal G, Calderon S, Anavi Y ( 2012). The dynamics of oral lichen planus: a retrospective clinico-pathological study. Head Neck Pathol.

[40] Peng Q, Zhang J, Ye X, Zhou G (2017). Tumor-like microenvironment in oral lichen planus: evidence of malignant transformation?. Expet Rev Clin Immunol.

